# Differential Gene Expression and Methylation Analysis of Melanoma in TCGA Database to Further Study the Expression Pattern of KYNU in Melanoma

**DOI:** 10.3390/jpm12081209

**Published:** 2022-07-25

**Authors:** Min Wang, Meng Liu, Yingjian Huang, Ziyang Wang, Yuqian Wang, Ke He, Ruimin Bai, Tingyi Ying, Yan Zheng

**Affiliations:** Department of Dermatology, The Second Affiliated Hospital, School of Medicine, Xi’an Jiaotong University, Xi’an 710004, China; mireille72wmin@163.com (M.W.); dr_liumeng@126.com (M.L.); yingjianhuang0418@163.com (Y.H.); ziyangwang0820@163.com (Z.W.); gtdywyq@163.com (Y.W.); 15332463230@163.com (K.H.); bai_ruimin@126.com (R.B.); yinty77@163.com (T.Y.)

**Keywords:** TCGA, melanoma, tumor target factors, KYNU, IL-10

## Abstract

Background: The aim of this study was to analyze and compare melanoma gene expression profiles in TCGA database through the application of different genes to explore the pathogenesis of melanoma. Furthermore, we confirmed the extent of the role of KYNU in melanoma and whether it can be a potential target for the diagnosis and treatment of melanoma. Methods: The gene expression profiles of melanoma samples were downloaded from TCGA database, and matrix files were synthesized to screen differential genes. The Kyoto Encyclopedia of Genes and Genomes (KEGG) signaling pathway analysis and GCDA broad institute were used to analyze common gene locus mutations and expression changes in melanoma, as well as methylation. In addition, the expression patterns of KYNU in melanoma were quantified by immunohistochemistry, Western blotting, qRT-PCR, software such as GEO DataSets and the Human Protein Atlas, and meta-analysis of skin diseases. KYNU was overexpressed in keratinocytes (HaCaT and HEKα) and melanoma cells (A375 and H1205-lu). CFDA-SE, Annexin V–PI double staining, and PI single staining were used to investigate the mechanism of KYNU in melanoma and its effects on melanoma proliferation, apoptosis, invasion, and migration. Results: The main signaling pathways involved in melanoma were EGF/EGFR–RAS–BRAF–MEK–ERK–CyclinD1/CDK4, Ras–PI3K–PTEN–PKB/AKT, and p14/p16 (CDKN2A)–MDM2–p53–p21–cyclinD1/CDK4/6–Rb/E2F. Moreover, *MITF*, *KIT*, *CDH1*. *NRAS*, *AKT1*, *EGFR*, *TP53*, *KIT*, and *CDK4* were elevated in melanoma, whereas *PTEN*, *cAMP*, and *BCL2* were reduced in melanoma. The copy number of tumor-promoting genes increased, while the copy number of tumor suppressor genes decreased. Changes in the copy number of the above tumor genes enriched in chromosomes were found through SNP gene mutations. The genes whose expression was negatively regulated by DNA methylation in melanoma included *KRT18*, *CDK2*, *JAK3*, *BCL2*, *MITF*, *MET*, *CXCL10*, *EGF*, *SOX10*, *SOCS3*, and *KIT*. The mutation rate of *KYNU* was high according to TCGA database. The KYNU level was decreased in melanoma. Overexpression of KYNU can promote changes in apoptotic BCL-2, metabolic KYN, 3-HAA, invasion and migration MMP9, E-cadherin, and other related proteins in melanoma. Fluorescence staining and flow analysis showed that a slower proliferation rate led to a stronger fluorescence intensity. In melanoma tumor cells with a low expression of KYNU, overexpression of KYNU could promote tumor cell apoptosis. IL-10 induced immunoregulatory changes in melanoma. The expression of MMP9 and AMPK decreased in A375, but the change in BCL-2 was not obvious. The expression of BCL-2 decreased significantly in H1205-lu. A375 showed cell-cycle arrest, indicating that IL-10 could slow down the cell cycle of melanoma. Conclusions: These results provide insights into the pathologic mechanisms of melanoma target genes and KYNU as a biomarker and potential therapeutic factor for melanoma.

## 1. Introduction

Melanoma is one of the most malignant skin tumors, which mainly includes four histopathological types, superficial spreading melanoma, nodular melanoma, lentigo maligna melanoma, and acral lentiginous melanoma [1]. About 25% of melanoma cases occur on pre-existing moles. Melanocytes are neural crest-derived cells that are mainly distributed in the basal epidermis, hair follicles, mucosal surfaces, meninges, and choroidal layers of the eye [2]. Approximately 1–8% of patients with a history of melanoma will develop multiple primary melanomas [3]. The transformation of melanocytes into malignant melanoma involves interactions among genetic factors, ultraviolet radiation (UV), and the tumor microenvironment. UV radiation mediates direct DNA damage by forming photoproducts and generating reactive oxygen species (ROS) [4]. Most melanomas develop through an initial phase known as the radial growth phase (RGP), including in situ and minimally invasive tumors, with a near 100% chance of cure. Despite the current shift toward early identification of melanoma, approximately 70% of melanomas have evolved by diagnosis to a point known as the vertical growth phase (VGP) or tumorigenic melanoma [5]. Pathogenic features of melanoma include heterotypic melanocyte growth and expansion, self-sufficient growth factors, insensitivity to growth inhibitors, evasion of apoptosis, potential for unlimited replication, ongoing angiogenesis, and tissue invasion and transfer [6].

Accurate classification helps patients get early diagnosis and the best possible treatment. The diagnosis of melanoma is mainly based on its clinical features. The ABCD rule is used to identify pigmented lesions suspected of early melanoma: asymmetry, border irregular, color inhomogeneous, and diameter greater than 5 mm [7]. Through a deep neural learning network, combined with the location of the disease and the classification characteristics of benign and malignant skin lesions, an algorithm can be used to identify benign and malignant skin lesions of epidermis and melanocyte origin from images of skin lesions [8]. Types of melanomas include desmoplastic melanoma, polypoid melanoma, primary dermal melanoma, verrucous malignant melanoma, pigmented epithelioid melanocytoma, mucosal melanoma, follicular melanoma, and melanoma with nonmelanocytic differentiation. Two subtypes of melanoma that are difficult to diagnose are nevoid melanoma and amelanotic melanoma [9]. Oral melanoma is extremely rare, accounting for 0.2% to 8.0% of all malignant melanomas, with the most common sites being the hard palate and gums [10]. Primary intestinal melanomas are rare, whereas small intestinal metastatic melanomas are common because cutaneous melanomas tend to metastasize to the gastrointestinal tract [11].

Proteins for clinical pathology of melanoma are related to epithelial–mesenchymal transition (SPARC and N-cadherin), cell adhesion and migration (ALCAM and ADAM-10), regulation of mitosis (PLK1), cell survival (FOXP1), and the function of the Golgi apparatus (GOLPH3 and GP73) [12]. A single gene mutation in the hair color gene *STX17G* causes an unbalanced behavior of melanocytes, leading to gray tendencies and the development of vitiligo and melanoma. The hair color genes *ASIP* and *MC1RE* can be based on black > jujube > sorrel, increasing the possible risk of melanoma [13]. The interactions between melanoma cells and their interactions with cell types in the microenvironment occur through endocrine and paracrine communication, direct cell–cell and cell–matrix contacts, and gap junctional intercell communication (GJIC) [14].

The positive rate of immunohistochemistry-related antigens of malignant melanoma can be queried through the DaKa pathological diagnostic tool (http://dia.dakapath.com/wap (accessed on 13 May 2021)). Among them, the almost always positive (≥95%) category includes CD146, IRF-4, MLH1, MSH-2, Myosin, ProExC, S100, SOX10, VERSICAN, and Vimentin; the usually positive (≥75%, <95%) category includes α-1-anti-trypsin, BCL-2, BCL-x, Cathepsin-K, CD63, HMB45, iNOS, KBA62, melan-A, Nestin, P16, PTEN, topo2A, and Tyrosinase; the frequently positive (≥55%, <75%) category includes α-1-anti-chymotrypsin, CD45RO, CD68, E-cadherin, MITF, MSE, PNL2, SOX2, TGF-β1, and XRCC6; the sometimes positive (≥35%, <55%) category includes BRAF-V600E, CD10, CD117, CD133, CD271, CD7, CEA-P, MAGE-1, N-Cadherin, Osteonectin, P21, P27, P504s, P53, PGP9.5, VEGF, WT-1, and XRCC5; the occasionally positive (≥15%, <35%) category includes CD138, CD5, CD64, CD99, Cyclin-D1, Inhibin, Iysozyme, MAGE-A4, mdm2, and VEGFr2; the rarely positive (≥5%, <15%) category includes Calretinin, CD56, CD57, EpCAM, FKBP12, FL1-1, GAP43, HBME-1, Hep-Par1, and Synaptophysin; the almost always negative (<5%) category includes 34bE12, actin-HHF-35, AE1, AE1/AE3, Arginase, Brachyuny, CA15-3, CA19-9, CAM5.2, CD15, CD163, CD19, CD1a, CD2, CD20, CD3, CD30, CD31, CD34, CD4, CD45, CD74, CD75, CD79a, CD8, CDX-2, CEA-M, CgA, CK20, CD7, CK8, CK-HMW, CK-LMW, GCDFP-15, GFAP, HCAD, HCG-α, HER2, Laminin, MAC387, Mesothelin, Myogenin, NY-ESO-1, OCT4, Osteocalcin, P63, PAX5, PAX8, PKK1, PSA, SMA, TAG72, TDT, and TFE3. Different patients have different clinical manifestations and pathological manifestations of melanoma, and the corresponding antigen markers should be selected for diagnosis and classification according to the medical history and antigen-related pathological characteristics.

Clark invasive grade and Breslow thickness grade are the current methods to judge the prognosis of melanoma. Breslow’s original study found that melanomas less than 0.76 mm thick had a good prognosis and no metastases [15]. Surgery remains the current gold standard. Extensive resection is performed according to the Breslow depth of the lesion and sentinel lymph nodes. Medium-depth melanomas (1.0 to 4.0 mm) should be considered for sentinel lymph node biopsy, and melanomas 0.75 to 1.0 mm in depth with high-risk lesions (ulcers and high mitotic index) should be considered for nodule biopsy [16]. For the mitotic rate (MR), the seventh edition of the TNM staging uses ≥1/mm^2^ as the main criterion for defining pT1b stage melanoma [17]. The surgical margins with Breslow thickness ≤2 mm are expanded to 1 cm beyond the normal skin lesions, and the margins with Breslow thickness >2 mm and ≤4 mm are expanded to 2 cm. Thicker skin lesions should also be expanded to at least 2 cm [18]. Prophylactic adjuvant regional perfusion for primary melanoma is currently in clinical trials. Postoperative radiotherapy, molecular targeted therapy, and other adjuvant methods should be considered in multiple melanoma and metastatic melanoma. Radiation therapy can be used for palliative treatment of inoperable patients combined with palliative adjuvant therapy such as hyperthermia, or nonradical resection of skin lesions can be performed after surgery [18]. Multivariate analysis indicated that the most important factors for patients with stage I and II melanoma were tumor thickness, the presence of ulcers, and the anatomical location of the primary tumor (head and neck lesions had a worse prognosis than extremity lesions). For patients with stage III melanoma, all of the above factors plus the degree of lymph node disease have prognostic significance. For patients with stage IV melanoma, the main prognostic factors are the number of metastatic lesions and the site of metastatic involvement (visceral lesions have a poorer prognosis) [19].

For molecular targeted therapy, melanoma-related target sites need to be sought. A recent study evaluated anti-CTLA-4 and anti-PD-1/PD-L1 blockade therapy in mucosal melanoma [20]. *KIT*-mutant melanomas exhibit clinical responses to inhibitors of type III transmembrane receptor tyrosine kinases [21]. Activating mutations in *c-KIT* are frequently found in mucosal melanoma, and this immunotherapy has emerged as a potential treatment. Both acral and mucosal melanomas have *KIT* mutations in approximately 15% of cases. *BRAF* or *NRAS* mutations are found in approximately 10–15% of acral melanomas, but the incidence of these mutations is lower in mucosal melanomas. Concomitant use of BRAF and MEK inhibitors is one of the standards of care for patients with advanced *BRAF*-mutant melanoma [22]. Combination strategies of BRAF inhibitors with ipilimumab and anti-CTLA-4 antibodies with MEK inhibitors or metformin are now in clinical trials [4]. Some important signaling pathways involved in the pathogenesis of melanoma are the mitogen-activated protein kinase (MAPK) pathway, the phosphoinositide 3-kinase (PI3K/PTEN/AKT) pathway, and the MITF signaling pathway. Currently important targeted therapies include vemurafenib, dabrafenib, and trametinib, as well as immunotherapies such as pembrolizumab, nivolumab, ipilimumab [6]. Dacarbazine chemotherapy remains the preferred drug for disseminated melanoma, but remissions are usually transient [23].

The above classification of melanoma, pathological features, diagnostic staging criteria, and surgical indications are used to improve the research on the diagnosis rate and therapeutic targets of melanoma. In this paper, by collecting skin melanoma-related data in TCGA database, we analyzed the changes and differential expression of melanoma-related genes. According to the data analyzed in TCGA database, the occurrence of melanoma involves many verified mutated genes, resulting in the occurrence of tumor-promoting activity, loss of tumor-suppressive function, and epigenetic modifications such as methylation to regulate gene expression [24]. TCGA database shows a high mutation rate of *KYNU* in melanoma. Studies have shown that PEG-KYNUase drugs can deplete the accumulation of the immunosuppressive product kynurenine (KYN) induced by its upstream metabolic enzyme indoleamine 2,3-dioxygenase (IDO) to treat B16-F10 melanoma in mice [25]. KYNU, as a hydrolytic enzyme in tryptophan metabolic pathway, is expressed in different cell types and diseases throughout the body. By verifying the expression of KYNU in melanoma, the role of KYNU in abnormal proliferative diseases was further reflected. We determined whether the expression pattern of KYNU in melanocyte-derived heterotypic tumor cells is the same as that in epidermal keratinocytes, whether the effects of KYNU on proliferation, differentiation, and metabolism are involved in the process of malignant transformation in melanoma, such as the process of melanogenesis, and whether mutations in the active site of the KYNU enzyme can serve as screening targets in the pathogenesis of melanoma. Thus, this paper takes KYNU as the research target to explore the expression changes of melanoma tumor cells in the functions of proliferation, differentiation, apoptosis, invasion, and migration.

## 2. Materials and Methods

### 2.1. Data Download and Analysis

TCGA is a cancer genomics program that provides publicly available data contributing to cutting-edge cancer research (https://portal.gdc.cancer.gov (accessed on 10 September 2020)). The copy numbers of gene transcriptomes in 472 samples of nevi and melanoma in the Exploration-Melanoma TCGA-SKCM project were collected, including three solid tissue normal samples (paracancerous), 100 primary melanoma samples, and the remaining 367 metastatic melanoma samples. The distribution of most frequently mutated genes is automatically generated by TCGA analysis website. The seq.counts.gv file of the gene expression data of the case was downloaded, and the R language and python language were used to splicing the count file into a genome-wide multi-sample matrix file with the gene symbol as the row and the clinical sample as the column. The merged matrix python file script can be found at https://github.com/wmin-debug/TCGA-melanoma-merge-matrix-python-file.git (accessed on 19 May 2022). Case-specific clinical data were also downloaded. The gene symbol and corresponding gene name were used to obtain the gene expression files of each sample. The gene expression averages of normal samples and melanoma samples were obtained through Excel and sorted, and the changes in differentially expressed genes were screened out.

The genome ID was converted to gene symbol through the Ensembl database, and then the differential gene expression levels of adjacent normal tissues and tumor tissues were compared. Since multiple gene variants were present, the expression changes of target genes were screened according to the type of disease. According to the melanoma-related tumor factors reported in the literature and the melanoma-related signaling pathway map05218 melanoma file in the KEGG database (https://www.genome.jp/kegg-bin/show_pathway?hsa05218+H00038 (accessed on 15 September 2020)), we searched for its upstream and downstream mechanisms and detected their relative expression abundance in normal tissues and tumors. By searching the melanoma-related genes in TCGA database, it was found that the proportion of copy number variation (CNV) among samples varied at different levels according to the upregulation and downregulation of copy number.

GCDA correlation analysis software (http://gdac.broadinstitute.org/runs/info/Mutation_Significance.svg (accessed on 6 February 2021)) was used to identify melanoma data of SKCM in TCGA database. Using the CopyNumberLowPass_Gistic2.Level and Correlate_Methylation_vs_mRNA.mage-tab functions, common gene mutation sites associated with melanoma could be obtained, in addition to the strength of the association between melanoma gene methylation and the impact on gene expression.

KYNU or the target disease-related gene list was entered through the online skin disease meta-analysis visualization tool (http://pathways-pellegrini.mcdb.ucla.edu/goTeles/dot_plot.html (accessed on 15 September 2020)) to obtain the relative expression levels of genes in various skin diseases.

The Human Protein Atlas online tool was used to query the expression levels of target gene KYNU (https://www.proteinatlas.org/ENSG00000115919-KYNU/tissue/metabolic/Phenylalanine%2C+tyrosine+and+tryptophan+biosynthesis (accessed on 20 September 2020)) in different tissues and cell types.

The GSE dataset was downloaded from NCBI GEO DataSets (https://www.ncbi.nlm.nih.gov/gds/ (accessed on 17 November 2020)), and the corrected FPKM data of the genomic expression levels in the GSE152699 and GSE152722 datasets were collected. GSE152699 is a melanoma cell sample, with *n* = 6 in the untreated group and vemurafenib-treated group; the mouse tissue samples in the GSE152722 dataset have *n* = 8 in the primary group, *n* = 13 in the repressed BRAF group, and *n* = 8 in the recurrent group. The expression level of KYNU was selected, and the expression difference of KYNU in melanoma was analyzed by *t*-test or one-way ANOVA.

### 2.2. Immunohistochemistry

All specimens were fixed with 10% paraformaldehyde solution for 24–48 h. Then, the tissue samples were dehydrated and embedded in paraffin. Here, 10 μm was selected as the target slice thickness for the wax block, and it was quickly adsorbed onto the glass. The sections were dewaxed by xylene twice and were soaked in a gradient concentration of ethanol for rehydration. Next, 3% H_2_O_2_ and 0.01 mol/L citrate buffer were used for antigen retrieval. After boiling the slices and then cooling down naturally, normal goat serum blocking solution was added dropwise. Then, 50 µL of the primary antibody with a dilution of 1:100–1:1000 was added at 4 °C overnight. Isotype IgG staining of the target antibody was set up, whereby 40–50 μL of biotinylated secondary antibody was added dropwise at 4 °C for 1 h. The sample was washed three times with PBS for 5 min. The DAB color was developed, and hematoxylin was counterstained. Ethanol along a concentration gradient and xylene were used to dehydrate and seal the slices, respectively.

KYNU staining was performed by immunohistochemistry in the same manner as described above. The intradermal nevus and melanoma tissue staining antibody was obtained from Sigma (WH0008942M2, 1:1000, 1 μg/mL dilution); the staining antibody of KYNU in psoriasis, melanoma, squamous cell carcinoma, and other diseases was obtained from GeneTex (#GTX33291, 1:200 dilution).

The semiquantitative analysis method of immunohistochemical results involved randomly selecting five high-power fields with a scale bar = 90 µm for each tissue sample of melanoma and intradermal nevus, followed by counting 100 cells in each field. According to the 1–4 grade classification, the staining intensity and the rate of positive cytoplasmic staining in each visual field were evaluated, and the scores of each group were classified into melanoma and intradermal nevus. The unpaired *t*-test was used to compare the statistical differences in the staining intensity and the rate of positive staining between the two groups.

### 2.3. Cell Culture and Transfection

Immortalized keratinocyte cell lines HaCaT and HEKα and human melanoma cell lines A375 and H1205-lu were obtained from an ATCC agent (Shanghai, China) or donated by the laboratory research group. Keratinocyte cell lines and melanoma cell lines were cultured and passaged in Dulbecco’s modified Eagle’s high-glucose medium (H-DMEM) containing 10% fetal bovine serum and 1% penicillin/streptomycin, before incubating at 37 °C with 5% CO_2_.

For KYNU overexpression, 1 μg of kynuORF-Pcmv66-Entry recombinant plasmid (OriGene, #RC214932) was mixed with 3 μL of PEI transfection reagent, and incubated with opti-MEM for 20 min for cell transfection. When the cells grew well and the cell fusion rate reached 50–60%, the cells were replaced with serum-free opti-MEM and incubated at 37 °C for 1 h in advance. Then, 200 μL/well of the mixed solution was evenly added dropwise into the six-well plate and incubated at 37 °C after mixing.

### 2.4. Western Blotting

When KYNU was overexpressed, the protein expressions of melanoma-related signaling pathways AKT and ERK1/2, metabolism-related signaling pathway AMPK, invasion and migration-related factors MMP2 and MMP9, and apoptosis-related factor BCL2 were detected by Western blot. The experimental method was as previously described [26]. Antibodies used in this paper were as follows: KYNU (GeneTex, #33291), AKT antibody (CST, #4691), ERK1/2 antibody (Santa Cruz Biotechnology, Shanghai, China, sc-514302), AMPK antibody (sc-398861; CST, #5832), p -AMPK (CST, #50081), MMP2 (CST, #4022), MMP9 (sc-21733), BCL-2 (CST, #15071), and β-actin (CST, #8H10D10).

### 2.5. qRT-PCR

The mRNA expression levels of E-cadherin, AKT and ERK1/2 were examined in keratinocytes (HaCaT and HEKα) and melanoma cells (H1205-lu and A375) by qRT-PCR. The cells were stimulated with IL-10 (40 ng/mL) and IFN-γ (40 ng/mL) in keratinocytes (HaCaT and HEKα) and melanoma cells (H1205-lu and A375) for 24 h, respectively. Cells were collected to extract total cell mRNA, and qRT-PCR was used to detect the mRNA expression of cell-cycle-dependent proteins and inhibitors. The relevant primers’ information of detected targets is shown in Table 1.

### 2.6. CFDA-SE Method to Detect Cell Proliferation

In keratinocytes (HaCaT and HEKα) and melanoma cells (H1205-lu and A375), the fluorescence intensity of cell proliferation was detected by fluorescent staining according to the protocol of the CFDA-SE staining kit (Beyotime, Shanghai, China, C0051). According to the cell proliferation state and density, the cells were evenly spread in six-well plates, and the cell confluence rate reached 50% after 8 h of adherence. Then, 20 mL of 10× CFDA-SE reagent cell labeling solution was diluted with PBS to 200 mL of 1× labeling solution. Next, 1 mL of CFDA-SE dye was dissolved at 1000× in the dark and stored at −80 °C. Then, 100 µL of 1000× dye was diluted to 10 mL of 10× dye, which was subsequently diluted to 1×, 2×, 5× and 10× dye concentrations, and then the cells were pre-stained to detect dye activity and toxicity toward the cells. The cell culture medium was removed, and then 1 mL of CFDA-SE dye was added to the cells for labeling for 10–15 min, followed by 1 mL of normal cell culture medium, before incubating for 15 min to continue staining. After removing the dye, the medium was replaced with normal medium, and the staining was observed with a green fluorescence microscope excited at 485 nm. It was found that the cells were marked by an increase in the staining concentration, and the cell viability decreased slightly at 10×. Finally, 5× was selected as the concentration for cell staining. The cells were cultured for 48–72 h, during which the dye overflowed and decayed, and the change in medium led to a change in the dye concentration. After 62 h of cell culture, the medium was removed, and the staining images of cell proliferation were collected under a fluorescence microscope. The cells were collected and analyzed by flow cytometry for the staining fluorescence intensity excited at 488 nm in different cell lines and treatment groups.

### 2.7. Annexin V–PI Double Staining to Detect Cell Apoptosis

Cells were stained in keratinocytes (HaCaT and HEKα) and melanoma cells (A375) using the Annexin V–PI double-staining detection kit (BD pharmingen, Shanghai, China, #556547). At least 10^6^ cells were digested, collected by centrifugation, and resuspended in 100 µL of medium. First, 5 µL of Annexin V was added to incubate for 10 min, and then 5 µL of PI was added for staining, before storing on ice or at 4 °C in the dark; the apoptosis rate was detected within 1 h.

Similarly, the above cells were stimulated with IL-10 (40 ng/mL) for 24 h, and the cell-cycle changes were detected using a PI single-staining kit (solarbio, CA1510). At least 10^6^ cells were collected and incubated with 500 µL of precooled 70% ethanol at 4 °C for 2 h to fix the cells; after centrifugation, the cells were washed with PBS or medium, resuspended with 100 µL of RnaseA solution, and incubated at 37 °C for 30 min. PI staining solution was added and incubated in the dark at 4 °C for 30 min for flow analysis of cell-cycle changes.

## 3. Results

### 3.1. Research on the Expression of Melanoma-Related Genes and Signaling Pathways in TCGA Database

**Figure 1:**
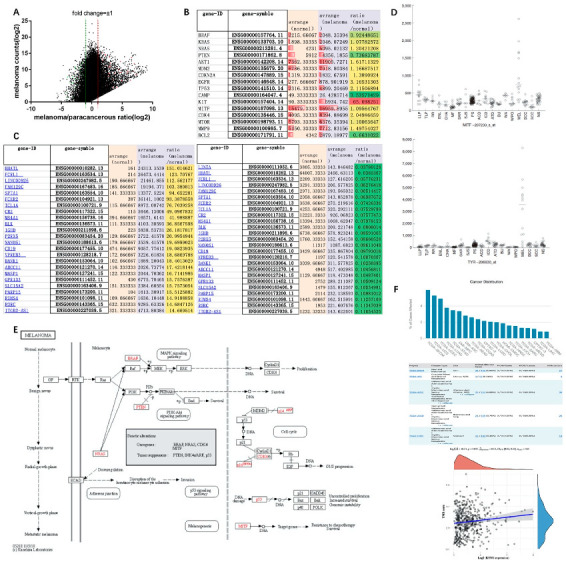
Screening of differentially expressed genes and signaling pathway mechanism of melanoma in TCGA database: (**A**) tumor-associated gene expression changes in melanoma samples from TCGA database; (**B**) gene expression of melanoma marker genes in TCGA database; (**C**) top 25 genes with significantly increased or decreased expression in melanoma compared with solid tissue normal samples after screening in TCGA database; (**D**) the expression levels of MITF and TRY are specifically elevated in melanoma; (**E**) melanoma signaling pathways in the KEGG database; (**F**) mutation distribution of KYNU in tumors from TCGA database.

Genome-wide expression revealed that there were significant changes in the differential expression of genes in melanoma tissue and paracancerous tissue. Some genes had very low expression levels in paracancerous tissues, but were significantly elevated in tumors, resulting in a significant increase in their differential fold changes, whereas some genes had high expression levels in both paracancerous and melanoma tissues, resulting in insignificant differential fold changes. We used the function log(express_average,2) to conform the data to a normal distribution using the gene expression value and the differential fold change, taking ±1 as the threshold (Figure 1A). Melanoma-related tumor genes *NRAS*, *AKT1*, *EGFR*, *TP53*, *KIT*, and *CDK4* could be observed in tumor tissues with high expression. The average level of *KIT* in melanoma was 65-fold higher than that in adjacent normal tissues, whereas *PTEN*, *cAMP*, and *BCL2* were downregulated almost twofold (Figure 1B). After screening the differentially expressed genes in melanoma of TCGA database, keratins (S100, *SLCL3A3*, etc.) were significantly increased, whereas Fc fragments (*FCRL1*, *FCER2*) and B-cell-related genes (*CD19*, *BLK*, etc.) were significantly decreased (Figure 1C).

According to the melanoma signaling pathway map in the KEGG database, oncogenes including *BRAF*, *NRAS*, *CDK4*, and *MITF*, and tumor suppressor genes including *p53*, *PTEN*, and *INK4a/ARF* (cyclin-dependent kinase inhibitor 2A, *CDKN2A*) could be identified [27]. The Ras–Raf–MAPK/PI3K–AKT signaling pathway, as a common signaling pathway in melanoma, promotes tumor proliferation and survival by regulating CDK4 and cyclinD1 [28]. PTEN inhibits tumor formation by reversing PI3K formation and preventing AKT activation [29]. p53 inhibits the G1/S cycle process by inhibiting the signaling pathways of cyclins CDK4/6 and cyclinD1, while transcriptional repressor Rb and transcription factor E2F are also involved in cycle signaling, and the MDM2 proto-oncogene can inhibit the expression of p53 (Figure 1E). In addition, RTK (EGFR), E-cadherin (CDH1), and MITF are also involved in the transformation of normal melanocytes to benign moles, atypical hyperplasia moles, and melanoma. EGFR, as an epidermal growth factor receptor, is involved in epidermal post-traumatic repair. When combined with epidermal growth factor EGF, it can initiate the expression of related genes in the nucleus. The correlation analysis with the Ras signaling pathway in melanoma can be combined with the pathological characteristics of melanoma at different stages to establish Cox regression analysis [30]. As a member of the cadherin family, E-cadherin (CDH1) is associated with cell adhesion. Downregulation of E-cadherin in melanoma can reduce the adhesion between epidermal keratinocytes and melanocytes. It can increase the invasion and migration of tumor cells [31]. MITF promotes tumor cell survival by targeting target genes to make them resistant to chemotherapy. In a multi-dermatology meta-analysis study, MITF and TRY were also found to be specifically elevated in melanoma (Figure 1D) [32,33]. However, TRY can be involved in the metabolism of tyrosinase in melanocytes to generate melanin. Whether it is a pathogenic gene of melanoma remains to be studied. Furthermore, the mutation rate of *KYNU* gene is higher in skin tumors than in other tissues, especially in melanoma, which has a mutation rate of 5.96%, involving 27 mutation sites changes in 455 amino acids. The tumor mutational burden (TMB) refers to the total number of substitutions and insertion/deletion mutations (somatic mutations) in the coding region of the assessed gene in the tumor cell genome. Spearman correlation analysis of TMB and KYNU gene expression showed that they were positively correlated in melanoma, with TMB scores of 2.5–5 mut/Mb. (Figure 1F). Therefore, the role of KYNU in melanoma is worth exploring. In the melanocyte signaling pathway [34], the MAPK and PI3K–AKT signaling pathways mediated by high levels of cAMP in cells are involved in the formation of melanocytes, while c-KIT is involved in the Ras-related signaling pathway, which is also an important research targets for melanocytes.

### 3.2. Study of the Mutated Genes and Loci of Melanoma in TCGA Database

**Figure 2:**
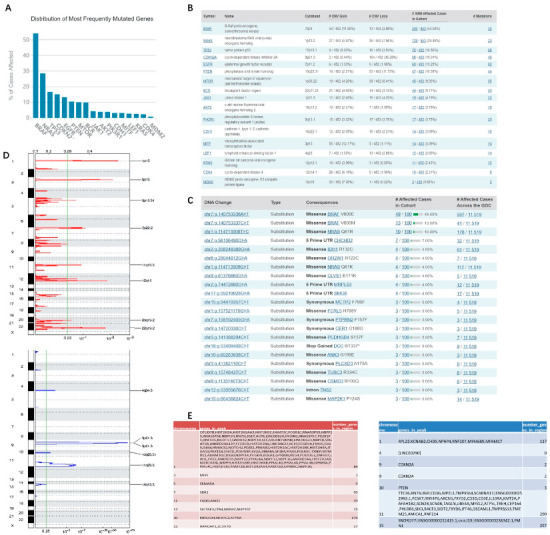
Most commonly mutated genes and mutation sites in melanoma: (**A**–**C**) melanoma tumor factor copy number variation and mutation sites in TCGA database; (**D**,**E**) GCDA broad institute analysis of melanoma tumor factor CNV in TCGA database. The *x*-axis represents the normalized upregulation (red) or downregulation signal (blue), and the green line represents the significance cutoff at Q = 0.25. In the table are the genes that are highly enriched on different chromosomes.

Similar to genes that were more abundantly expressed in melanoma, the mutated gene copy number variation (CNV) was also significantly elevated in melanoma (Figure 2A,B). Significant genes included *BRAF* (54/452, 11.95% copy number increased; 13/452, 2.88% copy number decreased), *NRAS* (27/452, 5.97% increased; 36/452, 7.96% decreased), *TP53* (16/452, 3.54% decreased), and *PTEN* (10/452, 2.21% increased; 53/452, 11.73% decreased). There are multiple mutation sites in the same gene, and *BRAF^V600E^* mutation is common in primary cutaneous melanoma, which features an A > T missense mutation caused by the base substitution of chromosome 7. About 50% of melanoma patients have mutations in this gene. Furthermore, 10% of patients have a T > C missense mutation of chromosome 1 *NRASQ61* (Figure 2C). For inoperable stage IIIC/IV *BRAF*-mutated melanoma, combined BRAF and MEK inhibitor therapy is the current gold standard [35]. Compared with cutaneous melanoma, the mutation rate of these two genes is lower in mucosal melanoma, and the corresponding *KIT* mutation rate is higher [36].

SNP studies in melanoma are valuable for precise diagnosis and therapy, which can facilitate medicinal chemistry to replace missing components and correct disturbed metabolic balance. According to the copy number variation caused by gene mutation in the GCDA broad institute, there are some common variation sites on the 24 chromosomes that are enriched for copy number variation genes. For example, chr3p13 showed an upregulated copy number of *MITF*, and chr12q15 showed an upregulated copy number of *MDM2*, while chr9p21.3 showed a downregulated copy number of *CDKN2A*, and chr10q23.21 showed a downregulated copy number of *PTEN* (Figure 2D,E).

### 3.3. DNA Methylation Studies of Melanoma in TCGA Database

DNA methylation is an important epigenetic modification that regulates the expression of adjacent sites. In the normal human genome, methylated CpG islands (abbreviated form of the deoxyribonucleotide “cytosine C–phosphate p–guanine G”) are located in the promoter and exon regions of genes. Methylation patterns refer to changes in gene and protein function by chemical modification of –CH_3_ to nucleic acids and proteins.

Current research suggests that N^6^-methylated adenine (m6A) is a well-studied methylation pattern. Adenine A directly forms a het-CH_3_ linkage to the N atom through the DNA/RNA methyltransferase *S*-adenosy l-methionine (SAM) as a methyl donor. For deoxyribocytosine (dC) in DNA, the methylation of bases at the C5 position is more complex. The C5 atom is not nucleophilic; thus, it cannot be directly methylated. DNA methyltransferases (DNMTs) undergo a 1,6-addition reaction with the aid of a sulfur-containing nucleophile (R–SH) to form a nucleophilic enamine substructure, which can subsequently be methylated by SAM cofactors. Importantly, R–SH is subsequently eliminated, thereby re-establishing the aromatic system to form the methylated base 5-methyl deoxycytidine (5mdC). This more complex enzymatic transformation enables nature to methylate non-nucleophilic carbon atoms, resulting in C–CH_3_ linkages with strong and stable C–C single bonds. Symmetrical methylation of CpG islands is a hallmark of silenced genes. Active DNMTs include Dnmt1, Dnmt3a, and Dnmt3b to activate deoxyribocytidine (dC). Dnmt1 maintains methylation during cell differentiation and regulates hemimethylation during DNA replication. That is, the template strand is methylated, and the newly synthesized strand still lacks methylation. Therefore, Dnmt1 epigenetically modifies the methylated dC (5mdC) during replication [37]. The DNA methylation inhibitor 5’-azacytidine (ZCyd), as a chromosomal breakage mutagen, can be activated into nucleoside triphosphate and incorporated into DNA and RNA. Incorporation of tRNA can inhibit tRNA methyltransferase, thereby interfering with the methylation and processing of tRNA, and further inhibiting the synthesis of DNA, RNA, and protein. However, ZdCyd is only incorporated into DNA, which is more efficient than Zcyd in inhibiting DNA methylation. Zcyd and ZdCyd enzymes have a pro-deamination effect, and the products are cytotoxic. ZdCyd is 10 times more toxic to cells and animals than Zcyd. The activity of the DNA methyltransferase Dnmt1 in mammals is reduced by the cytotoxicity of Zcyd and ZdCyd, which induces rapid demethylation within hours through its covalent binding to Zcyd at CpG hemimethylated sites [38]. The methods for detecting DNA methylation are DNA extraction and bisulfite-modified DNA pyrosequencing with the Epi Tech Fast Bisulfite kit (QIAGEN). DNA methylation levels can be detected by sequencing PCR, a MassARRAY system spectrometer, or a nanoDrop spectrophotometer.

**Figure 3:**
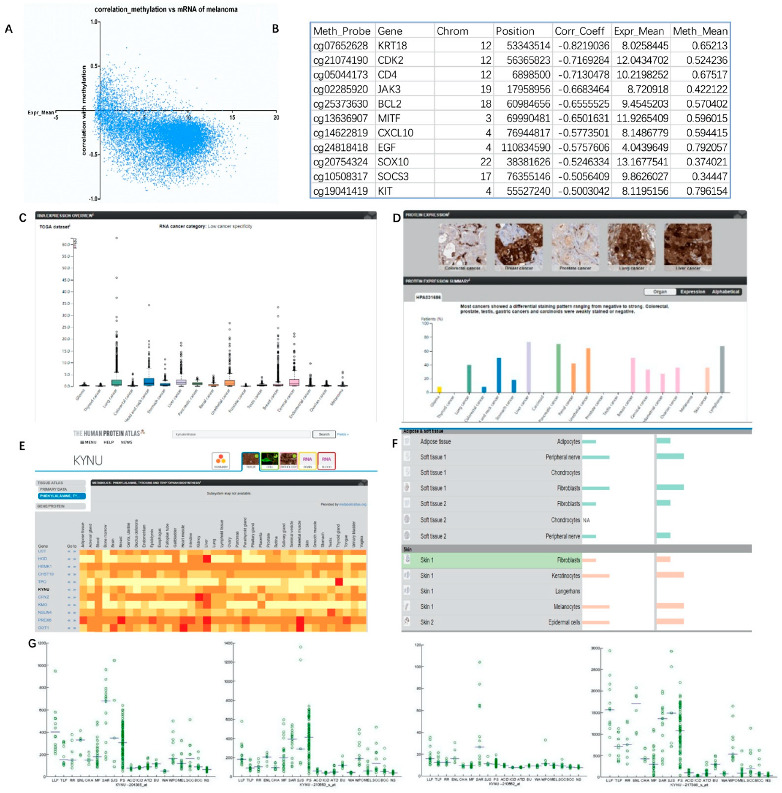
Levels of melanoma methylation indicating why KYNU could be a melanoma target: (**A**,**B**) GDCA broad institute analysis of the distribution of gene mRNA levels and DNA methylation associations in TCGA database melanoma; (**C**) mRNA levels of KYNU in different tumors from TCGA database; (**D**) protein level and immunohistochemical staining of KYNU in different tumors from TCGA database; (**E**) heatmap of protein expression of KYNU in different tissues; (**F**) relative expression distribution of KYNU in different kinds of skin cells; (**G**) expression distribution of KYNU in different probes/samples in meta-analysis of skin diseases.

In this experiment, the methylation analysis results of melanoma in TCGA database were obtained through the GDCA broad institute analysis tool. From the perspective of methylation level and gene expression distribution, methylation and gene expression were mostly negatively correlated, the correlation index was below −0.5, and the relative gene expression level was most distributed between 5 and 10 (Figure 3A). The degree of influence of melanoma methylation level on gene expression level was determined after sorting according to its correlation index. It was found that the methylation level of melanoma-related genes was strongly correlated with gene expression (Figure 3B and Supplementary Figure S1) as follows: *KRT18* (correlation coefficient = −0.8219), *CDK2* (corr_Coeff = −0.71693), *JAK3* (corr_Coeff = −0.66835), *BCL2* (corr_Coeff = −0.65555), *MITF* (corr_Coeff = −0.65016), *MET* (corr_Coeff = −0.59344), *CXCL10* (corr_Coeff = −0.57735), *EGF* (corr_Coeff = −0.57576), *SOX10* (corr_Coeff = −0.52463), *SOCS3* (corr_Coeff = −0.50564), and *KIT* (corr_Coeff = −0.5003).

### 3.4. Expression Level of KYNU in Melanoma

The data from TCGA database can reflect the expression level of KYNU in different tumors. Its mRNA expression level was high in lung cancer and liver cancer, but the expression level in melanoma was not significantly increased (Figure 3C,D). The Human Protein Atlas online tool can further analyze the expression level of KYNU in different diseases, different tissues, and cell types (Figure 3E). Similarly, data from the metabolome database reflected that KYNU was highly expressed at the tissue level in the liver, lung, lymph node, blood, and breast, but moderately expressed in skin. In various cells of the skin epidermis and dermal fat and soft tissue, KYNU was relatively highly expressed in keratinocytes, melanocytes, and fibroblasts, but was not significantly expressed in Langerhans cells and chondrocytes (Figure 3F). KYNU is expressed in a variety of skin diseases. It was verified by microarray in the literature [32] that the expression of KYNU is significantly increased in psoriasis (PS) and cutaneous sarcoidosis (SAR). However, moderate increases were seen in tumors such as melanoma (MEL), squamous cell carcinoma (SCC), and basal cell carcinoma (BCC) of the skin (Figure 3G).

**Figure 4:**
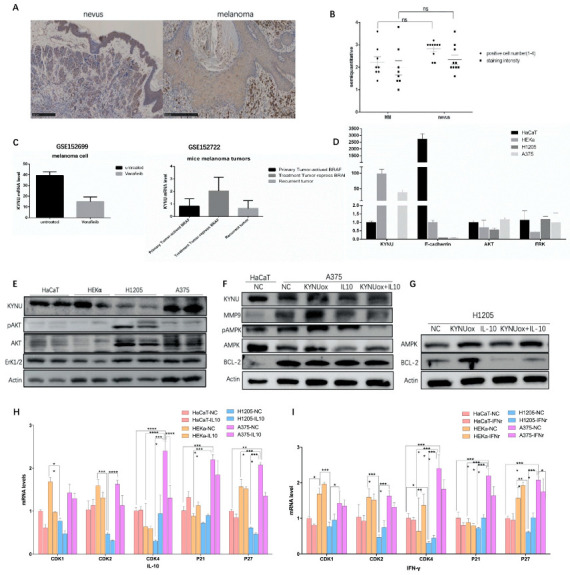
Expression of KYNU and KYNU regulatory factors in melanoma: (**A**,**B**) immunohistochemical staining of KYNU in intradermal nevi and melanoma patient tissues, melanoma *n* = 8, intradermal nevi *n* = 9, scale bar = 250 µm. Significance was determined by one-way ANOVA test, ns (*p* > 0.05); (**C**) changes in the expression of KYNU regulated by BRAF in melanoma cells and mouse melanoma tissues; (**D**,**E**) protein and mRNA expression levels of KYNU and signaling pathways in different keratinocyte and melanoma cell lines; (**F**,**G**) metabolism-, apoptosis-, and invasion-related protein changes in A375 and H1205-lu melanoma cells stimulated by KYNU overexpression or IL-10; (**H**,**I**) mRNA expression of cyclin-dependent protein kinases and inhibitors in HaCaT, HEKα, H1205-lu, and A375 cell lines induced by IL-10 and IFN-γ. * *p* < 0.05, ** *p* < 0.01, *** *p* < 0.001, and **** *p* < 0.0001 according to ordinary one-way ANOVA compared to controls.

Immunohistochemical analysis of KYNU protein expression was performed in different cells in benign intradermal nevi and melanoma. In intradermal nevus, the staining intensity of KYNU in keratinocytes was significantly higher than that in benign nevus cells. Nevus cells had a mature phenomenon and the expression of KYNU was uniform. In contrast, malignant melanoma cells had obvious nuclear atypia and increased melanogenesis, but no significant expression of KYNU was found in the nucleus. Non-keratinocytes were counted in the high-power field of view on the 90 µm scale, and the influence of melanin was removed. A semi-quantitative analysis of the staining depth and the high-power field-positive cell rate was performed using grades 1–4. The results showed that the staining intensity of KYNU expression in malignant melanoma cells was weaker than that in benign nevus cells, and the positive cell rate was reduced, but there was no significant difference (Figure 4A,B and Supplementary Figure S2A). This suggested that the expression of KYNU may be decreased during the transformation of nevus cells to malignant melanoma cells.

A parallel comparison of immunohistochemical staining of KYNU was conducted in different cells in skin inflammatory diseases and skin tumors. The results showed that KYNU was abundantly expressed in keratinocytes and inflammatory cells. Psoriatic skin lesions and lichen planus chronic dermatitis had a large number of inflammatory cells infiltrated in the superficial dermis, where KYNU hyperstaining could be seen. The keratinocytes of normal skin lesions were uniformly stained with KYNU, and the basal layer was stained more deeply in the high-power field. However, psoriatic keratinocytes stained less in parakeratinocytes. The proliferating keratinocytes had enlarged nuclei and a reduced cytoplasm, while most cells had KYNU-stained granules. Keratinocytes in precancerous lesions of Bowen’s disease and actinic keratosis (AK) showed atypical cells with enlarged nuclei, chromatin pyknosis, nuclear division, and decreased cytoplasmic expression of KYNU. AK also had abundant KYNU hyperstained inflammatory cell infiltration. Intraepidermal tumors of well-differentiated SCC in situ showed marked cellular atypia and decreased cytoplasmic KYNU expression. In well-differentiated SCC, the expression of KYNU was higher in the nonmalignant cells of the epidermis because the tumor invades from the epidermis to the deep layer. The tumor cells invading deep dermis had obvious atypia and decreased expression of KYNU, but some tumor cells were hyperchromatic. In poorly differentiated SCC, the morphological features of tumor cells were diverse, and most of the tumor cells were highly stained with KYNU. On the other hand, in tumor cells, during the progression of melanoma, excluding a large amount of melanin production, the relative expression of KYNU in tumor cells was decreased (Supplementary Figure S2B).

According to the above expression of KYNU in different cells of different diseases, the order of staining depth was inflammatory cells > keratinocytes > nevus cells > tumor cells. For the positive rate of KYNU cells in tumors, the order was keratinocytes > tumor cells, well-differentiated squamous cell keratinocytes > precancerous lesions Bowen, AK, and poorly differentiated SCC > well-differentiated SCC. Some tumor cells had a high positive rate of KYNU.

### 3.5. Study of the Regulatory Factors of KYNU in Melanoma

Collecting the GSE152699 and GSE152722 datasets, we found that KYNU expression decreased after treatment with the anti-BAF drug vemurafenib in melanoma cell lines. Upon mutation of the *BRAF^V600E^* locus in mouse tissues, the expression of KYNU decreased in the primary melanoma group (activate BRAF), increased in the dormant group (repress BRAF), and decreased in the recurrent group (escape dormant) (Figure 4C), indicating that KYNU is negatively correlated with BRAF activation. It was reported that the application of PEG-KYNUase overexpressing KYNU can be used to treat melanoma [25]. It is considered that KYNU can inhibit tumor growth by enhancing metabolism in tumors and reducing the accumulation of the upstream immunosuppressive product kynurenine.

Accelerated metabolism of melanoma tumors can lead to a decline in survival. Generally, glucose metabolism can accelerate tumor growth. Whether KYNU-mediated tryptophan metabolism is involved in melanoma metabolism is worth discussing. Metabolic secretion can be used to detect whether melanoma invasion and migration are promoted. For example, in the glioma mouse model, KYNU was significantly increased in different models as detected by alpha-[^11^C]-methyl-l-tryptophan (AMT)-PET. Immunohistochemical verification showed that KYNU was deeply stained in gliomas [39]. In melanoma, the effect of pigment granules on KYNU immunohistochemical staining should be considered. Therefore, the changes in expression levels in tumors were detected by protein quantification.

Two keratinocyte cell lines (HaCaT and HEKα) and two melanoma cell lines (H1205-lu and A375) were selected. It was found that KYNU was significantly expressed in keratinocyte cells, while the expression of KYNU was significantly decreased in the melanoma cell line H1205-lu and significantly increased in A375. Compared with keratinocytes, H1205-lu proliferated slowly, while A375 proliferated more quickly with increased apoptosis. The adhesion force of melanoma cells is very weak; hence, it was necessary to further study whether KYNU had a regulatory effect on the proliferation, differentiation, apoptosis, adhesion, invasion, and migration of melanoma cells. The expression of AKT in melanoma signaling pathway was significantly increased in melanoma cell lines, and the phosphorylation of AKT in H1205-lu was also significantly increased. The ERK1/2 pathway signaling was not altered significantly, with weak increases in the melanoma cell lines (Figure 4D).

The mRNA expression of KYNU in melanoma cells was similar to the expression pattern of the protein in the cells. The expression of KYNU in the quickly proliferating A375 cells was significantly increased, while the expression in the slowly proliferating H1205-lu cells was significantly decreased. The expression of E-cadherin in melanoma cells with low adhesion was significantly lower than that in keratinocytes. It is considered that the adhesion of melanoma cells is weakened to promote its invasion and metastasis in skin tissue. The mRNA expression changes of signaling pathways AKT and ERK1/2 in melanoma were weaker than their changes at the protein level (Figure 4E).

### 3.6. Effects of KYNU on Proliferation, Differentiation, Apoptosis, Invasion, and Migration of Melanoma

In the process of tumor cell proliferation and differentiation, calcium, a common differentiation inducer, can promote the terminal differential expression of P63 in the basal layer of keratinocytes. When induced by exogenous low calcium, the expression of miR-203 was low and the expression of P63 was high. At high calcium, miR-203 expression was high and p63 expression was low [40]. In the process of increasing the concentration of extracellular anhydrous CaCl_2_ from 0.09 mM to 1.2 mM (up to 2.8 mM), cell differentiation was enhanced, and synthesis decreased. Calcium induces changes in intracellular and extracellular calcium concentrations within a short period of time. Desmosomes are formed for 5 min, which are symmetrical and functional for 2–5 h. This creates epidermal mechanical tension and may also provide signaling complexes for keratinocyte differentiation. The calcium concentration of the skin epidermis increases gradually from the basal layer to the spinous layer and the granular layer, but decreases in the stratum corneum. These changes can promote the expression of differentiation-related indicators at each layer, e.g., keratin 5/14 in the basal layer, keratin 1/10, Involucrin, and transglutaminase in the spinous layer, and loricrin and Filaggrin in the granular layer to the stratum corneum [41]. Buffering of intracellular calcium prevents terminal cell differentiation, which is an indicator of early and late differentiation. Other intracellular ion concentrations have corresponding effects on keratinocytes; for example, K^+^ activates enzyme activity, Ca^+^ enhances cell permeability and adhesion, and Zn^+^ deficiency induces enteropathic acrodermatitis. The adhesion molecules between cells and between cells and the extracellular matrix include the integrin family, the immunoglobulin superfamily, the selectin family, and the cadherin family. As a member of the cadherin family, E-cadherin stains the epidermal cell membrane [42], and it can also participate in the invasion and migration of melanoma cells.

Matrix metalloproteinases MMP2 and MMP9 can induce the degradation of extracellular matrix components, promoting tumor cell infiltration and spread in the bloodstream [2]. The expression of invasion factor MMP9 was significantly higher in A375 than in HaCaT. AMPK is a factor that regulates the homeostasis of energy metabolism in cells, and its expression in A375 was attenuated compared with HaCaT. Apoptotic factor BCL-2 was significantly higher in A375 than in HaCaT (Figure 4F). After overexpression of KYNU, the expression of MMP9 and AMPK was significantly increased in A375, while the expression of BCL-2 was significantly increased in H1205-lu (Figure 4F,G). According to previous studies, 3-HAA, a downstream product of KYNU, may activate Th1 apoptosis through caspases, thereby inducing Th1/Th2 balance imbalance and apoptosis.

Immunosuppressive regulation in tumors also plays an important role. Some studies found that IFN-γ promotes the production of IDO, an upstream enzyme of the KYNU metabolic pathway, thereby promoting tumorigenesis. The IFN-γ–JAK1/JAK2–STAT1/STAT2/STAT3–IRF1 axis mainly regulates PD-L1 expression [43]. As an anti-inflammatory factor, IL-10 at a high concentration may also play a role in immune tolerance and, thus, act as a tumor suppressor. B cells suppress immune responses primarily by producing IL-10 [44]. The production of IFN-β and IL-10 synergistically induces IDO in mesenchymal stem cells through the STAT1 pathway [45,46]. By stimulating melanoma cells with IL-10 (20 ng/mL), it was found that the expression of AMPK was significantly decreased in A375 and H1205-lu cells, while the expression of BCL-2 was also significantly increased in H1205-lu cells (Figure 4F,G).

### 3.7. CFDA-SE Cell Staining and Flow Cytometry to Detect Cell Proliferation

The above protein quantification reflected the changes in tumor factors such as cell proliferation, apoptosis, invasion, and migration in melanoma. Furthermore, cell proliferation, apoptosis, and cycle changes can be visually observed by fluorescently labeling cells, immunofluorescence microscopy, and flow cytometry.

The commonly used methods to detect cell proliferation include CCK8/MTT cell staining, microplate readers, absorbance detection, [^3^H]-thymidine incorporation, and cell proliferation marker labeling. Nuclear markers related to cell proliferation include B-edu, PNCA, and Ki67. When carrying out antibody staining, cells are usually fixed to observe the expression state of cells at the same time point. Assuming that the binding fluorescent group is modified to the chromosome in the nucleus, it is shown that the weak toxicity weakens the effect on cell division, and its fluorescence is halved with the division of the progeny. This can be analyzed by flow or fluorescence microscope, and the cell proliferation passage can be reflected by multiple peaks of fluorescence, allowing more detailed cell proliferation information to be obtained.

According to the literature, CFDA-SE is a highly liposoluble fluorescent dye that can easily enter cells and can irreversibly bind to intracellular amino acids. CFDA-SE cell staining was used to culture the cells for 72 h. The CFDA-coupled intracellular protein excited green fluorescence at 488 nm fluorescence, which could enter the daughter cells during cell division. At the same time, the fluorescence intensity also became half that of the parent cell.

**Figure 5:**
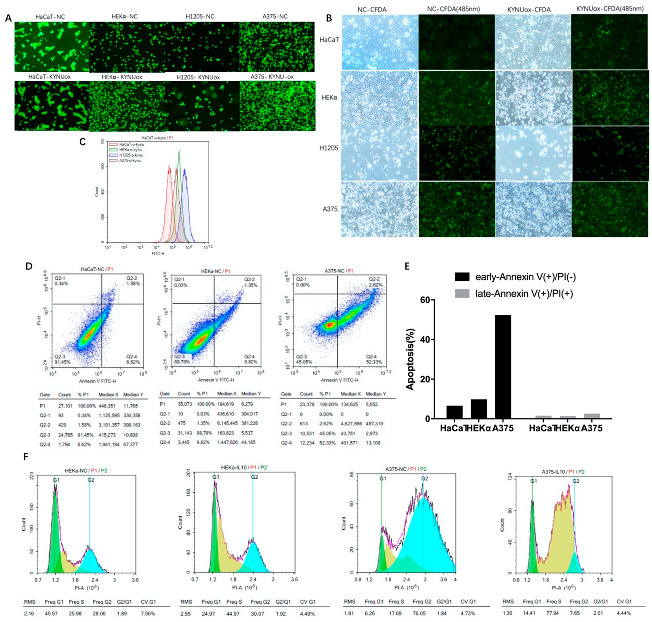
Effects of KYNU overexpression on proliferation and apoptosis of melanoma cells or keratinocytes and the effects on melanoma cell cycle of IL-10 and IFN-γ: (**A**–**C**) cells were stained with CFDA-SE after 8 h of adherence and continued to culture for 62 h. Immunofluorescence and flow cytometry were used to detect changes in the peak fluorescence of cells; (**D**,**E**) apoptosis flow analysis of Annexin V–PI double staining in keratinocytes and melanoma cell lines; (**F**) PI single-stained cell-cycle flow analysis in normal and IL-10-stimulated HEKα and A375 cells.

After the cells adhered for 8 h, keratinocytes and melanoma cells were labeled with a concentration gradient of CFDA for 30 min. The morphology of HaCaT and HEKα cells was regular after dye endocytosis, while the brittle H1205-lu and A375 cells became round after dye endocytosis (Figure 5A). Fluorescence spillover was observed during cell culture. After 62 h of culture, according to the microscope slide without CFDA-SE dye-treated cells, the total rate of cell proliferation decreased after KYNU overexpression, the number of HEKα apoptotic cells increased significantly, and H1205-lu cells proliferated slowly (Figure 5B). The fluorescence of cells treated with CFDA-SE dye was reduced, while the staining fluorescence was in the form of a single peak according to flow cytometry. After overexpression of KYNU, there was no obvious change in the fluorescence intensity of cells, but the fluorescence was stronger in H1205-lu cells, which proliferated slowly. Therefore, fluorescence was effluxed during cell culture, and the fluorescence decreased as the cells proliferated (Figure 5C).

### 3.8. Cell-Cycle and Apoptosis Detection in Melanoma

Apoptotic cells in keratinocytes and melanoma cells were detected by flow cytometry. It was found that A375 had significantly more early apoptotic cells than keratinocytes (Figure 5D,E), which was consistent with the previous Western blot results showing that BCL-2 apoptotic protein was increased in melanoma.

Furthermore, cyclin-dependent kinases (CDKs) and cyclin-dependent kinases inhibitors (CDKIs) were used to detect the immune function of keratinocytes and melanoma cells. Cyclical changes are induced by the regulated inflammatory factors IL-10 and IFN-γ. Through the G0–G1 restriction point R point, CDK synthesizes DNA replication-related proteins and enzymes in late G1 phase and checks chromosomes before DNA synthesis. The cyclinD–CDK2/4/5/6 complex has a transition role through the G0–G1 R restriction site. The R restriction point is controlled by the Rb pathway (p16/INK4A–CDK4/CDK6–CyclinD1–Rb). The cyclinE–CDK2 complex regulates the G1–S phase transition, and the cyclinA–CDK2 complex regulates the transition through the S phase. CDKIs include the Kip/Cip family (P21 WAF/Cip1, P27 Kip1, and P57 Kip2) that bind to inhibit most cyclin–CDK complexes. The INK4 family of proteins (P16, P15, P18, and P19) are specific inhibitors of cycD–CDK4 and cycE–CDK2, and they bind to CDK monomers. The tumor suppressor gene P53 can cause DNA damage-induced cell-cycle arrest and inhibit cycD1–CDK4 and cycE–CDK2. P27 (CDKN1B) regulates serum removal, contact inhibition, and cell-cycle arrest caused by TGF-β [47].

The cytokine IL-10 (40 ng/mL)-stimulated CDK1 in keratinocytes (HaCaT and HEKα) decreased significantly. The expressions of CDKs and CDKIs in H1205-lu were lower, and the changes were not obvious. In A375, the expression of CDK2 and CDK4 decreased, while the expression of its inhibitors P21 and P27 also decreased, leading to cell-cycle arrest (Figure 4H). According to the cell cycle analyzed by flow cytometry after PI staining, HEKα cells were stimulated with IL-10 (40 ng/mL) and shifted to the right, and the proportion of cells in the S–G2 phase was significantly higher than that of the cells in the G1 phase. However, after stimulation with IL-10 in A375 cells, the cell cycle shifted to the left, and a large number of cells were in the S phase instead of the G2 phase (Figure 5F). The abovementioned regulation of cell-cycle-dependent proteins by IL-10 shows that IL-10 could slow down the cell cycle of melanoma A375 cells. However, the cell cycle did not change significantly in HaCaT when stimulated by IFN-γ (40 ng/mL), whereas the expression of CDK4 and P27 increased in HEKα, and the cell cycle expression increased slightly in H1205-lu. In A375, the cell-cycle expression decreased, and the changes in CDK4 and P27 were more obvious (Figure 4I).

### 3.9. 3D and Coculture Cell Models Established to Analyze the Feasibility of the KYNU Tyrosinase Locus as a Melanoma Target

The tumor cells were inoculated into the solidified medium, and the 0.4–0.8% agarose solidified medium and the nanostructured hydrogel were added with nutrients and the extracellular matrix required by the cultured cells in normal body tissues. This allows better simulating the proliferation and migration state of the cells in the body, and the culture time can be extended to observe the differentiation state of the cells [48]. For example, abnormally well-differentiated or poorly differentiated tumor cells are formed between skin basal cells and basal progenitor cells, and M5 cytokines induce psoriasis model keratinocytes with accelerated cell proliferation and reduced differentiation indicators. The model of coculture of melanocytes and keratinocytes was successfully established, and the transport process of melanosomes could be observed [49]. Furthermore, tyrosine kinase metabolism is involved in melanogenesis in melanocytes. In the catalytic metabolism of KYNU, the first step of catalytic metabolism is formed through the proton transfer of the tyrosine site Try275 in the active center of the enzyme. Therefore, it is considered whether mutation of the KYNU tyrosine site in melanocytes is involved in the occurrence of melanoma, which can be used to construct a melanoma model and an endogenous site for melanoma screening.

## 4. Discussion

In this paper, the genome-wide analysis of melanoma tumor samples in TCGA database found that signaling pathways in melanoma mainly involved the EGF/EGFR–RAS–BRAF–MEK–ERK–CyclinD1/CDK4, Ras–PI3K–PTEN–PKB/AKT, cell-cycle pathway p14/p16 (CDKN2A)–MDM2–p53–p21–cyclinD1/CDK4/6–Rb/E2F, melanogenesis-related *MITF*, *KIT*, cell adhesion *CDH1*, and other genes. The basal expression of *TP53*, *AKT1*, *EGFR*, *KIT*, and *CDK4* was high in cells and increased in melanoma cells. Furthermore, the expression levels of genes such as *PTEN*, *cAMP*, and *BCL2* were generally decreased in melanoma cells. According to the copy number variation of genes with higher expression abundance in melanoma, oncogenes *BRAF* and *NRAS* showed more upregulation than downregulation, while tumor suppressor genes *TP53* and *PTEN* showed a greater downregulation ratio. Through the study of SNP gene mutation sites in melanoma, it was found that *BRAF^V600E^* had a mutation rate of more than 50%, while 10% of patients had a T > C missense mutation of chromosome 1 *NRASQ61*. Chr3p13 enriched on chromosomes showed an increased copy number of *MITF*, while chr12q15 showed an increased copy number of *MDM2*. On the other hand, chr9p21.3 showed a decrease in the copy number of *CDKN2A*, while chr10q23.21 showed a decrease in the copy number of *PTEN*. For the negative regulation of gene expression by DNA methylation, *KRT18*, *CDK2*, *JAK3*, *BCL2*, *MITF*, *MET*, *CXCL10*, *EGF*, *SOX10*, *SOCS3*, and *KIT* could be seen in melanoma. In summary, we analyzed the changes in gene expression levels in melanoma, the roles of genes in melanoma signaling pathways, mutation sites, and DNA methylation modifications. The related gene expression was measured at the genome-wide level, and the genes that played a role in the transformation of melanoma from benign moles to malignant were identified, thus providing predictive targets for disease classification and treatment.

The pathogenesis of KYNU in melanoma was explored through the expression changes of KYNU in melanoma. As a therapeutic target for melanoma, BRAF can regulate the expression of KYNU. The expression level of KYNU in melanoma tissues and cells was quantitatively analyzed by immunohistochemistry and Western blot, and it was found that the expression level of KYNU in melanoma was lower. Overexpression of KYNU could promote changes in apoptosis-, metabolism-, invasion-, and migration-related proteins in melanoma. In the quickly proliferating melanoma A375 cell line, the expression levels of apoptotic protein BCL-2 and invasion-related MMP9 were significantly increased, while KYNU could promote the increased expression of MMP9 and AMPK. However, in the H1205-lu cell line with a slower proliferation rate, the expression levels of KYNU and other related proteins were lower, and the overexpression of KYNU significantly increased the expression levels of BCL-2 and AMPK. The expression of KYNU was significantly increased in HEKα and A375, but lower in H1205-lu. The expression of endogenous KYNU and overexpression of KYNU were observed through CFDA-SE fluorescent staining of cells. Overexpression of KYNU showed that the number of H1205-lu and HEKα proliferating cells was significantly reduced, the number of apoptotic cells was significantly increased, and the apoptosis of A375 was obvious. According to the change in fluorescence intensity between cells by flow analysis, a slower cell proliferation rate resulted in a stronger fluorescence intensity. Therefore, the proliferation rate of HaCaT was fastest, followed by A375 and HEKα, while that of H1205-lu was slowest. In conclusion, when the expression level of KYNU in tumor cells is high, the overexpression of KYNU has no significant effect. Knockout of KYNU can accelerate the cell cycle and promote tumorigenesis. In melanomas with low expression of KYNU, overexpression of KYNU can promote tumor cell apoptosis. This is considered to be related to the decreased expression of its upstream immunosuppressive product KYN and the increased downstream expression of 3-HAA. On the other hand, immunomodulatory therapy in melanoma is currently an important tumor therapy method, e.g., anti-CD4 and anti-PD-L1 therapy, which are used for clinical validation. Previous studies found that IFN-γ can induce the expression of IDO and promote tumorigenesis, while IL-10 plays an anti-inflammatory role in inflammation. In this experiment, the change in IL-10-induced melanoma protein expression was used to test whether it could be used as a tumor suppressor of melanoma. In melanoma cells (A375 and H1205-lu), the expression of MMP9 and AMPK decreased in A375 cells after stimulation with IL-10 (20 ng/mL), while BCL-2 did not change significantly. The expression of BCL-2 was significantly decreased in H1205-lu. When stimulated with IL-10 (40 ng/mL), CDK1 was significantly downregulated in HaCaT and HEKα. The expressions of CDKs and CDKIs in H1205-lu were lower, but the changes were not obvious. In A375, the expressions of CDK2 and CDK4 were downregulated, and their inhibitors P21 and P27 were also downregulated, resulting in cell-cycle arrest. When stimulated with IL-10 (40 ng/mL), the cell cycle of HEKα cells shifted to the right by PI staining, and the proportion of cells in the S–G2 phase was significantly higher than that of cells in the G1 phase. In A375 cells, however, the cell cycle shifted to the left, and a large number of cells were in the S phase rather than the G2 phase. The abovementioned regulation of cell-cycle-dependent proteins by IL-10 showed that IL-10 could slow down the cell cycle of melanoma A375 cells. For the differentiation and migration of melanoma cells, a change in calcium concentration inside and outside the cell in a short time may be involved in the process of cell differentiation, and its concentration varies in the different layers of keratinocytes. E-cadherin is expressed on the surface of cell membrane and mediates the adhesion between cells and the extracellular matrix. The expression of E-cadherin in melanoma cells is significantly lower than that in keratinocytes, and it can participate in the invasion and migration of tumor cells.

In this paper, by exploring the relevant indicators and cell changes in the process of melanoma proliferation, differentiation, apoptosis, invasion, and migration, the gene mutation types commonly involved in the abnormal differentiation of melanoma from melanocytes into primary melanoma and recurrent and metastatic melanoma were identified. The results of this article can be used reduce the occurrence and recurrence of melanoma cells by (1) reducing the heterotypic differentiation rate of tumor cells, (2) establishing directional differentiation to benign organized tissues, (3) enhancing tumor cell apoptosis, (4) slowing down the cell cycle, and (5) reducing the possibility of invasion and migration.

## Figures and Tables

**Table 1:** Primer sequences in this paper.

Detect Genes	Primer Sequence (5′–3′)
human GAPDH	forward 5′–TGTTGCCATCAATGACCCCTT–3′ reverse 5′–CTCCACGACGTACTCAGCG–3′
human KYNU	forward 5′–GTCACAACTACAACTTCACGGA–3′ reverse 5′–CCCCACTGAACAGGATCACTG–3′
human E-cadherin	forward 5′–AAAGGCCCATTTCCTAAAAACCT–3′ reverse 5′–TGCGTTCTCTCTATCCAGAGGCT–3′
human AKT1	forward 5′–CCAGCCTGGGTCAAAGAAGT–3′ reverse 5′–GTCCTCGGAGAACACACGTT–3′
human ERK1/2	forward 5′–CAGTTCTTGACCCCTGGTCC–3′ reverse 5′–AATGGGTGACACACACAGGG–3′
human CDK1	forward 5′–AAACTACAGGTCAAGTGGTAGCC–3′ reverse 5′–TCCTGCATAAGCACATCCTGA–3′
human CDK2	forward 5′–CCAGGAGTTACTTCTATGCCTGA–3′ reverse 5′–TTCATCCAGGGGAGGTACAAC–3′
human CDK4	forward 5′–ATGGCTACCTCTCGATATGAGC–3′ reverse 5′–CATTGGGGACTCTCACACTCT–3′
human P21	forward 5′–TGTCCGTCAGAACCCATGC–3′ reverse 5′–AAAGTCGAAGTTCCATCGCTC–3′
human P27	forward 5′–AACGTGCGAGTGTCTAACGG–3′ reverse 5′–CCCTCTAGGGGTTTGTGATTCT–3′

## Data Availability

Publicly available datasets were analyzed in this study. This data can be found according to link and accession number in methods. Complete original analysis data could be received through the author’s email.

## Supplementary Materials

The following supporting information can be downloaded at https://www.mdpi.com/article/10.3390/jpm12081209/s1: Figure S1. Overview of GDCA broadinstitute analysis of the effect of DNA methylation on gene expression levels in melanoma in the TCGA database; Figure S2. Immunohistochemical staining of KYNU in melanoma and different skin diseases.
