# Supplementary material for: Differential Gene Expression and Methylation Analysis of Melanoma in TCGA Database to Further Study the Expression Pattern of KYNU in Melanoma
Source: J Pers Med. 2022 Jul 25;12(8):1209. doi: 10.3390/jpm12081209 (PMC9329910; doi:10.3390/jpm12081209)
Supplement: Supplementary file 1 [file jpm-12-01209-s001.zip › supplementary figure S1.pdf]

Meth_Probe	Gene	Chrom	Position	Corr_Coeff	Pval	Qval	Expr_Mean	Meth_Mear
cg21171625	DNALI1	1	38022607	-0.905819	0.00E+00	0.00E+00	5.291415	0.570319
cg15518883	SIT1	9	35650561	-0.9026733	0.00E+00	0.00E+00	5.409007	0.732851
cg11578532	TSTD1	1	161008127	-0.9010815	0.00E+00	0.00E+00	5.212243	0.664942
cg07786657	CD247	1	167487633	-0.893683	0.00E+00	0.00E+00	6.14484	0.788342
cg20317872	DENND2D	1	111743202	-0.8861873	0.00E+00	0.00E+00	7.734231	0.671921
cg16068833	CD52	1	26644515	-0.8811514	0.00E+00	0.00E+00	7.879695	0.697562
cg02423817	PTPRCAP	11	67203661	-0.8780302	0.00E+00	0.00E+00	7.733273	0.721505
cg05462570	NAALADL1	11	64814948	-0.8727233	0.00E+00	0.00E+00	5.164327	0.409392
cg10402417	TBC1D10C	11	67171476	-0.8670866	0.00E+00	0.00E+00	6.509044	0.594206
cg21249754	PTPN6	12	7060206	-0.8629839	0.00E+00	0.00E+00	8.958638	0.705929
cg00903584	PTPN7	1	202128682	-0.8619511	0.00E+00	0.00E+00	7.591584	0.659616
cg08075204	BIN2	12	51718112	-0.8575301	0.00E+00	0.00E+00	7.077247	0.769915
cg25671438	ACAP1	17	7240223	-0.8559412	0.00E+00	0.00E+00	6.846298	0.572407
cg01305625	PDLIM4	5	131593812	-0.8517467	0.00E+00	0.00E+00	7.839426	0.699017
cg07682037	FERMT3	11	63974153	-0.8510041	0.00E+00	0.00E+00	9.671298	0.487351
cg16462073	LAT	16	28995843	-0.8440817	0.00E+00	0.00E+00	7.545465	0.785506
cg05564251	SP140	2	231090640	-0.8434221	1.62E-100	5.09E-101	6.232752	0.706194
cg07994485	FAM178B	2	97652440	-0.8426603	0.00E+00	0.00E+00	6.42017	0.670583
cg10673833	MYO1G	7	45018849	-0.8422825	0.00E+00	0.00E+00	7.408168	0.778664
cg02358862	CORO1A	16	30197211	-0.8411404	1.81E-99	5.71E-100	9.99403	0.693669
cg12947833	HSPB2	11	111783499	-0.8407895	0.00E+00	0.00E+00	7.297636	0.310399
cg01072786	GLB1L	2	220107847	-0.8387809	0.00E+00	0.00E+00	8.767008	0.589811
cg11692409	SERPINF1	17	1665181	-0.8365314	0.00E+00	0.00E+00	12.24383	0.550812
cg00032205	TSPYL5	8	98290372	-0.8353067	0.00E+00	0.00E+00	7.032563	0.55864
cg23612220	TNFAIP8L2	1	151129298	-0.8339776	0.00E+00	0.00E+00	6.178103	0.757709
cg17078393	LCK	1	32717002	-0.8324328	0.00E+00	0.00E+00	7.115752	0.807434
cg24674703	CD5	11	60869960	-0.830381	0.00E+00	0.00E+00	6.383773	0.745535
cg24459792	LAPTM5	1	31230846	-0.8286882	0.00E+00	0.00E+00	11.66512	0.723087
cg04881903	CAPG	2	85637116	-0.8285622	0.00E+00	0.00E+00	10.82851	0.614889
cg24691453	S100A4	1	153518384	-0.8275865	0.00E+00	0.00E+00	10.46994	0.496253
cg26769927	SPN	16	29674972	-0.8248027	0.00E+00	0.00E+00	7.358736	0.762055
cg02836135	SCML4	6	108052093	-0.8235695	0.00E+00	0.00E+00	6.065603	0.628737
cg06613515	PSTPIP1	15	77287656	-0.8230898	0.00E+00	0.00E+00	6.199011	0.617364
cg06133110	SCRN1	7	30028307	-0.8224482	0.00E+00	0.00E+00	10.23746	0.499891
cg23545105	OCIAD2	4	48908833	-0.8223265	0.00E+00	0.00E+00	6.347006	0.500679
cg07652628	KRT18	12	53343514	-0.8219036	0.00E+00	0.00E+00	8.025845	0.65213
cg00490406	AIM2	1	159046773	-0.8218924	0.00E+00	0.00E+00	6.134589	0.497512
cg15046675	CD37	19	49838777	-0.8211683	0.00E+00	0.00E+00	8.528701	0.700094
cg24612198	CD3E	11	118175631	-0.8208379	0.00E+00	0.00E+00	7.978214	0.827723
cg05656364	VAMP8	2	85804732	-0.820415	0.00E+00	0.00E+00	9.443289	0.438946
cg14110548	HCST	19	36393696	-0.8189727	0.00E+00	0.00E+00	7.267915	0.708115
cg17044311	ABCC2	10	101542983	-0.818435	0.00E+00	0.00E+00	6.693458	0.713504
cg06958535	LAX1	1	203734478	-0.8179124	0.00E+00	0.00E+00	4.614016	0.741215
cg04253799	LOC10012867	19	35597062	-0.8161649	0.00E+00	0.00E+00	4.319494	0.646931
cg07106761	ALDH2	12	112205145	-0.8159853	0.00E+00	0.00E+00	8.88957	0.629227
cg00328227	C1orf59	1	109204325	-0.8131289	0.00E+00	0.00E+00	7.214538	0.459665
cg09153080	CD6	11	60739183	-0.8124812	0.00E+00	0.00E+00	6.979674	0.684629
cg17467873	CMBL	5	10307544	-0.8110409	0.00E+00	0.00E+00	6.5693	0.326761
cg11550547	KCTD14	11	77734490	-0.8104747	0.00E+00	0.00E+00	4.813776	0.676986
cg17169196	LTA	6	31540026	-0.809266	0.00E+00	0.00E+00	3.494283	0.84414
cg19320294	CA14	1	150229871	-0.8089849	0.00E+00	0.00E+00	8.318369	0.492728
cg00219921	CD8A	2	87012810	-0.808227	0.00E+00	0.00E+00	7.844427	0.87038
cg03724640	CD27	12	6553145	-0.807404	0.00E+00	0.00E+00	7.279216	0.657599
cg11430077	GATA3	10	8099018	-0.8054242	0.00E+00	0.00E+00	5.101644	0.687822
cg13500819	MGC29506	5	138725400	-0.8048397	0.00E+00	0.00E+00	6.830932	0.756329
cg17771150	LCP1	13	46756209	-0.8048258	0.00E+00	0.00E+00	11.03459	0.630498
cg22066521	ABCB5	7	20687223	-0.8045287	0.00E+00	0.00E+00	7.730052	0.644789
cg02249390	LTB	6	31550612	-0.8037439	2.55E-84	8.03E-85	6.668835	0.718666
cg03684062	S1PR4	19	3179364	-0.8032124	0.00E+00	0.00E+00	5.472774	0.566719
cg01834022	EVC2	4	5710411	-0.8031126	0.00E+00	0.00E+00	6.899549	0.299389
cg17127769	LCP2	5	169724590	-0.8022931	0.00E+00	0.00E+00	9.023297	0.515521
cg02499214	RHOF	12	122230490	-0.8014694	0.00E+00	0.00E+00	6.816542	0.686029
cg07641284	C16orf54	16	29756951	-0.8004442	0.00E+00	0.00E+00	5.76901	0.662829
cg06970090	SPOCK2	10	73848823	-0.800199	0.00E+00	0.00E+00	8.987258	0.554648
cg27491050	TBC1D7	6	13326842	-0.7998875	0.00E+00	0.00E+00	10.64525	0.707095
cg21163717	DOK2	8	21769903	-0.7989706	0.00E+00	0.00E+00	7.472543	0.699587

Supplementary Figure S1. Overview of GDCA broadinstitute analysis of the effect of DNA methylation on gene expression levels in melanoma in the TCGA database.
