# Supplementary material for: Differential Gene Expression and Methylation Analysis of Melanoma in TCGA Database to Further Study the Expression Pattern of KYNU in Melanoma
Source: J Pers Med. 2022 Jul 25;12(8):1209. doi: 10.3390/jpm12081209 (PMC9329910; doi:10.3390/jpm12081209)
Supplement: Supplementary file 1 [file jpm-12-01209-s001.zip › supplementary figure S2.pdf]

A

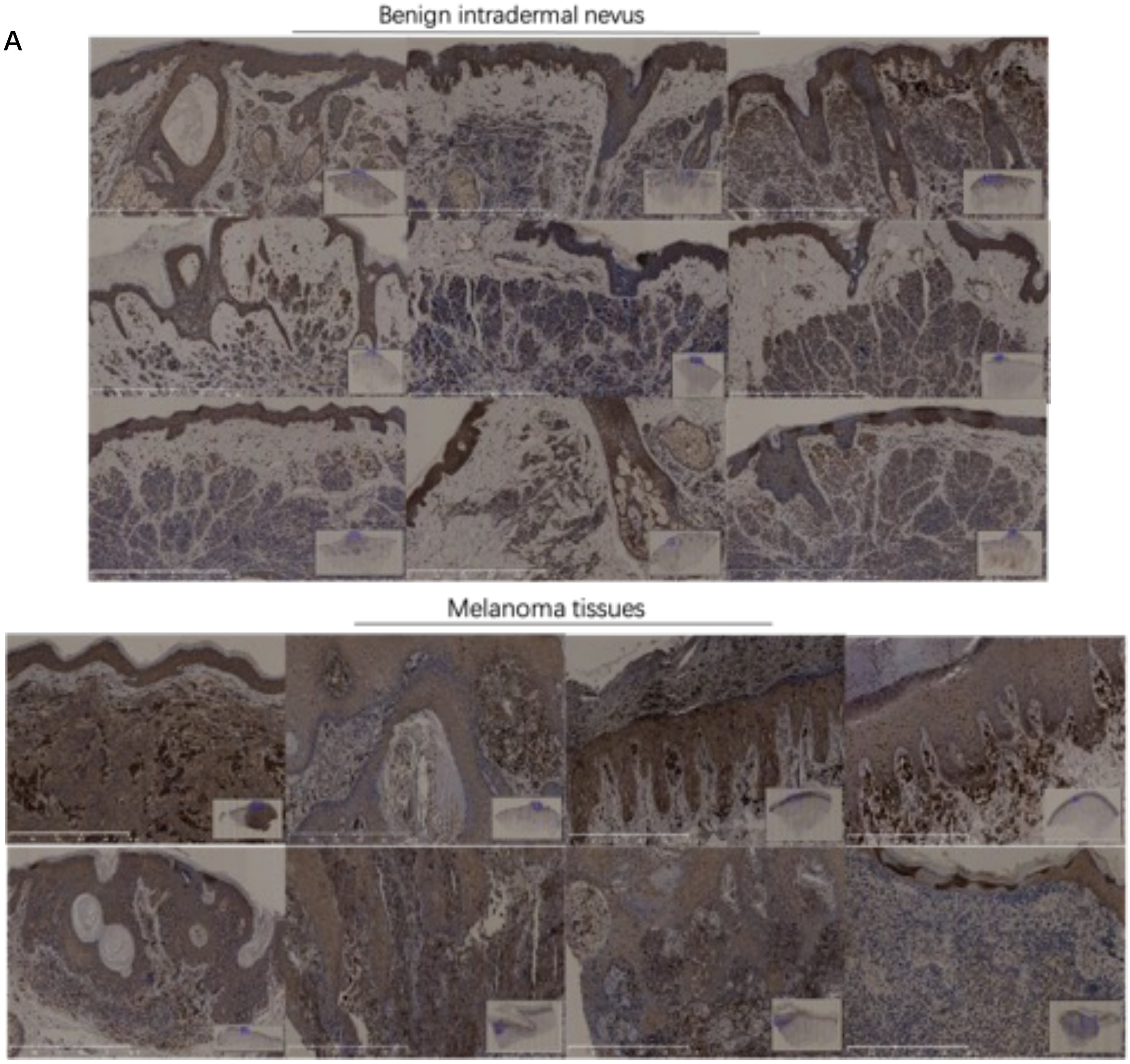

B

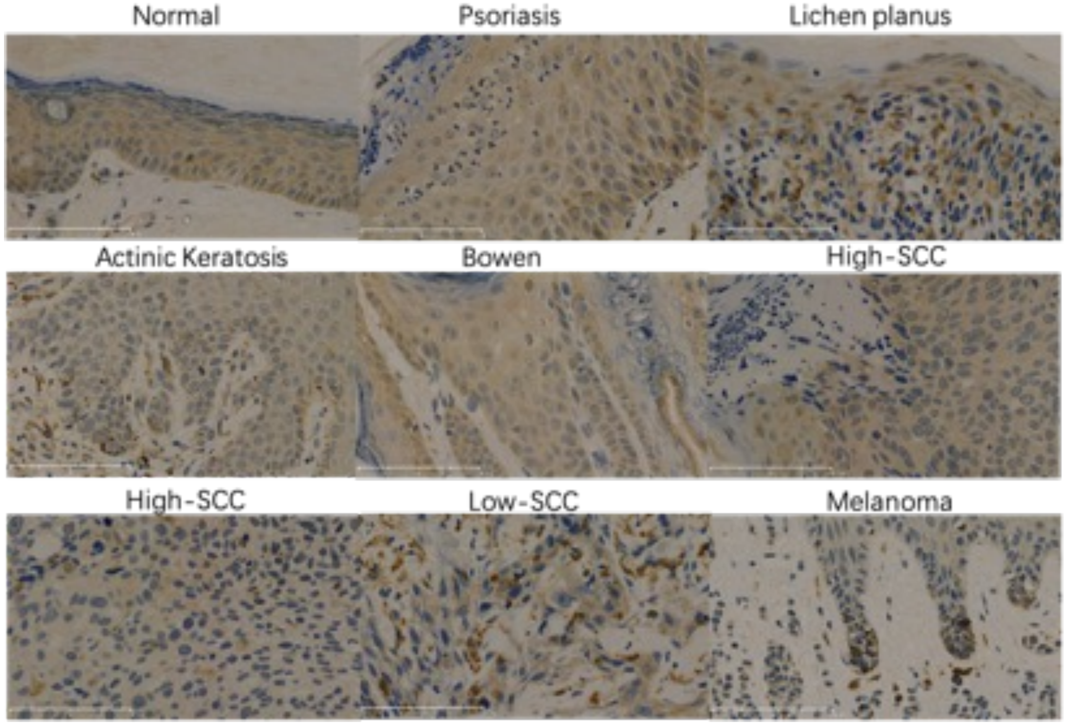

Supplementary Figure S2. Immunohistochemical staining of KYNU in melanoma and different skin diseases: (**A**)Immunohistochemical staining of KYNU in intradermal nevi and melanoma patient tissues, melanoma n=8, intradermal nevi n=9, Bar=500um; (**B**)Immunohistochemical staining of KYNU in various types of skin inflammatory diseases and skin tumors, each group n=3-4, Bar=100um.
